# Rotation of X-ray polarization in the glitches of a silicon crystal monochromator

**DOI:** 10.1107/S1600576716009183

**Published:** 2016-07-06

**Authors:** John P. Sutter, Roberto Boada, Daniel T. Bowron, Sergey A. Stepanov, Sofía Díaz-Moreno

**Affiliations:** a Diamond Light Source Ltd, Harwell Science and Innovation Campus, Didcot, Oxfordshire OX11 0DE, UK; b ISIS Neutron and Muon Source, Rutherford Appleton Laboratory, Didcot, Oxfordshire OX11 0QX, UK; c Advanced Photon Source, Argonne National Laboratory, Argonne, Illinois 60439, USA

**Keywords:** X-ray monochromator glitches, X-ray polarization, EXAFS

## Abstract

EXAFS studies on dilute samples are usually carried out by collecting the fluorescence yield using a large-area multi-element detector. This method is susceptible to the ‘glitches’ produced by all single-crystal monochromators. Glitches are sharp dips or spikes in the diffracted intensity at specific crystal orientations. If incorrectly compensated, they degrade the spectroscopic data. Normalization of the fluorescence signal by the incident flux alone is sometimes insufficient to compensate for the glitches. Measurements performed at the state-of-the-art wiggler beamline I20-scanning at Diamond Light Source have shown that the glitches alter the spatial distribution of the sample’s quasi-elastic X-ray scattering. Because glitches result from additional Bragg reflections, multiple-beam dynamical diffraction theory is necessary to understand their effects. Here, the glitches of the Si(111) four-bounce monochromator of I20-scanning just above the Ni *K* edge are associated with their Bragg reflections. A fitting procedure that treats coherent and Compton scattering is developed and applied to a sample of an extremely dilute (100 micromolal) aqueous solution of Ni(NO_3_)_2_. The depolarization of the wiggler X-ray beam out of the electron orbit is modeled. The fits achieve good agreement with the sample’s quasi-elastic scattering with just a few parameters. The X-ray polarization is rotated up to ±4.3° within the glitches, as predicted by dynamical diffraction. These results will help users normalize EXAFS data at glitches.

## Abstract 2

Rotation of X-ray polarization at the glitches of a monochromator composed of single crystals of silicon is observed. This effect can be explained by a model taking full account of the X-ray source, the effects of multiple-beam dynamical diffraction, and the coherent and Compton scattering from the sample.

## 1. Introduction   

**Figure 1:**
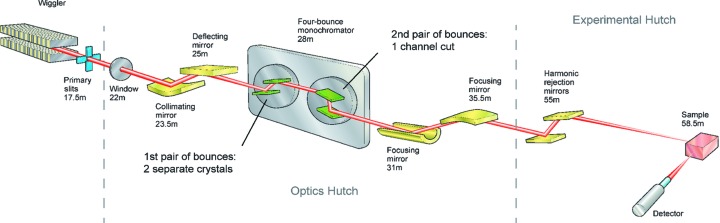
Layout of the I20-scanning beamline at the Diamond Light Source.

The Diamond Light Source synchrotron beamline I20 contains a wiggler that produces X-rays for the energy-scanning branch I20-scanning, which is displayed in Fig. 1 ▸. I20-scanning is dedicated to EXAFS experiments and has a number of special optical components for this purpose. The four-bounce silicon crystal monochromator, which will be the subject of this article, consists of two parallel but separate crystals on the first axis and a channel cut on the second axis. The overall configuration of the four Bragg reflections is 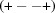. For energy scanning, the two axes rotate in opposite senses. This design is unusual, but it offers a fixed beam exit along with the advantage of angular acceptance and energy resolution that depend only on the Bragg reflection and are not broadened by the incident beam divergence. All crystals now in use are oriented to the symmetric 111 reflection, which allows the selection of X-rays with photon energies of 4–20 keV with a bandpass 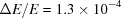. A wiggler with 24 periods at a period length of 83 mm was chosen as the source because it emits a continuous distribution of high spectral flux over a broad spectral range. The collimating mirror reduces the divergence of the wiggler beam so that as many photons as possible enter the monochromator within its angular acceptance, which being equal to the Darwin width (a few µrad) is very small. All together, the wiggler, the white-beam mirrors and the monochromator produce X-ray beams with higher flux and narrower bandwidth than would typically be found at many other EXAFS beamlines. The remaining optical components downstream from the monochromator are curved mirrors for focusing the X-rays onto the sample and flat mirrors for harmonic rejection. The experimental hutch therefore would ideally receive an X-ray beam of high flux (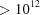
 photons per second at 10 keV) and low harmonic contamination focused into a 300 × 250 µm spot on the sample.

**Figure 2:**
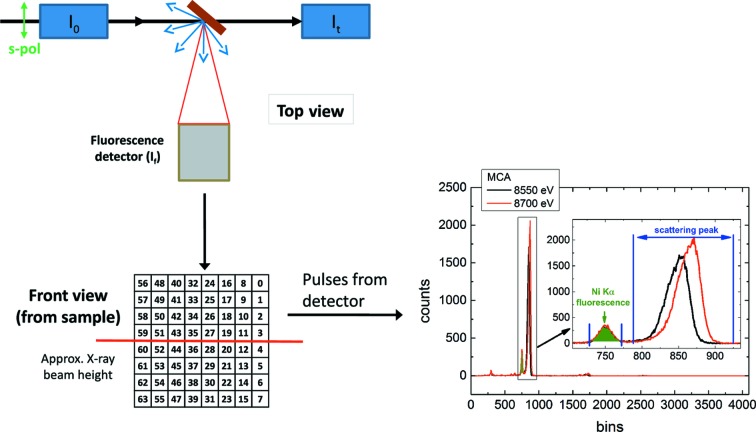
Schematic showing how fluorescence yield from the sample is collected and analyzed at I20-scanning. In the upper left, 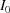, 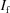 and 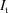 are, respectively, the incident, fluorescent and transmitted intensities at the sample. In the lower left is a drawing of the 64 pixel germanium solid-state detector. Each pixel has an area of 5 × 5 mm. In the lower right, the fluorescence photons in the detector are shown separated from non-fluorescent scattering according to energy by a multi-channel analyzer.

The special design of I20-scanning has allowed the performance of EXAFS measurements on samples as dilute as 10 micromolal (Bowron & Diaz-Moreno, 2014 ▸). Dilute samples cannot easily be examined by measuring their X-ray absorption as a function of incident photon energy in transmission because the absorption of the matrix dominates the EXAFS near the absorption edge of the absorber, although such studies have been successful for millimolar samples when great care was taken to remove systematic errors from the data collection (Chantler *et al.*, 2015 ▸; Islam *et al.*, 2015 ▸). Instead, the fluorescence yield of the absorber in the sample is measured using a large-area fluorescence detector. To reduce the non-fluorescent scattering background as much as possible, the detector is oriented facing the sample as shown in Fig. 1 ▸. The line from the sample to the detector is horizontal and rotated 90° from the path of the incident X-ray beam, which is mostly horizontally polarized. (In fact, the wiggler beam is slightly depolarized out of the horizontal plane, with effects that will be discussed in §2.) As described in Fig. 2 ▸, the detector is a 64 pixel germanium monolithic solid-state device with 5 × 5 mm pixels. The pulses from the detector are binned by a multi-channel analyzer according to their heights. This energy discrimination capability is crucial for separating the fluorescence photons to be measured from the non-fluorescent scattering that still does reach the detector.

**Figure 3:**
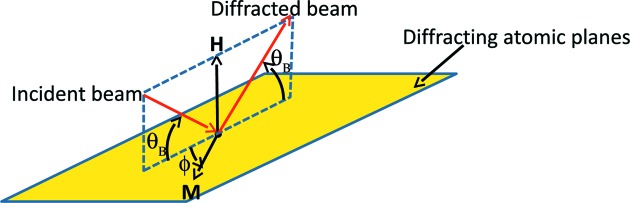
Relevant vectors and angles that determine energy settings at which a crystal’s Bragg reflection will display glitches: 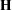, the reciprocal lattice vector associated with the diffracting atomic planes; 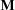, a vector perpendicular to 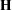 that is used as an azimuthal reference; ϕ, the azimuthal angle at which the crystal is cut; and 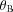, the Bragg angle.

**Figure 4:**
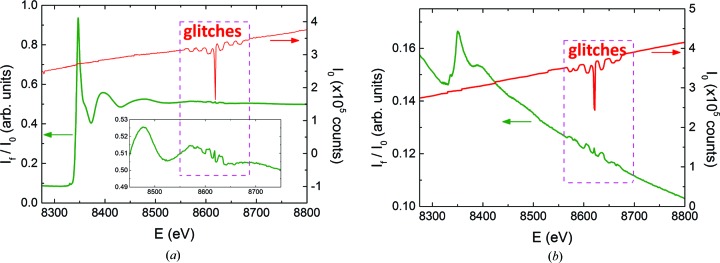
EXAFS results measured at I20-scanning around the Ni *K* absorption edge in aqueous solutions of nickel nitrate, 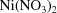, at concentrations of 1 millimolal (*a*) and 10 micromolal (*b*).

Normally the fluorescence count rate 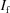 measured by the germanium detector is divided by the incident X-ray intensity, which in this case is monitored by the reading 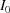 of an ionization chamber immediately upstream from the sample (see Fig. 2 ▸). In this way, fluctuations of the incident beam’s intensity caused by electron current decay, top-ups, thermal drift of the optics and so forth would usually be compensated. However, as increasingly dilute samples have come to be measured at I20-scanning, the usual normalization has failed more and more frequently at the sharp dips in flux, or ‘glitches’, that occur when the monochromator is tuned to select certain photon energies. Glitches are seen in energy scans of all crystal monochromators, and they appear more frequently as the selected photon energy increases. The Monochromator Crystal Glitch Library of the Stanford Synchrotron Radiation Lightsource (SSRL) shows a set of examples (Stanford Synchrotron Radiation Lightsource, 1999 ▸). The energy settings at which any crystal monochromator displays glitches depends on both 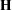, the reciprocal lattice vector normal to the diffracting atomic planes, and the azimuthal angle ϕ from some vector 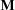 normal to 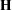 at which the crystal is cut. (See Fig. 3 ▸ for definitions.) Some efforts have been made to reduce the number of glitches by optimizing the monochromator crystals’ azimuthal cuts (Tang *et al.*, 2015 ▸). An attempt made at I20-scanning to apply the usual normalization procedure to a sample of aqueous nickel nitrate solution in the neighborhood of a glitch is shown in Fig. 4 ▸. There it is clear that the glitches are not in fact fully compensated in the 1 millimolal solution, and that in the 10 micromolal solution the glitches introduce features into the normalized data that are as strong as the EXAFS oscillations one would wish to measure. As the sample grows more dilute, the glitches increasingly distort the spline to be selected for post-edge data processing, making it impossible to reliably extract the EXAFS signal. One may note that the pixels of the germanium detector, being single crystals themselves, can also introduce glitches into the measurements. However, according to Kirkland *et al.* (1988 ▸), silicon PIN photodiode detectors used in transmission EXAFS generate output spikes of the order of only 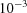 of the X-ray absorption edge step. They also state that these spikes will have negligible effects on fluorescence EXAFS measurements because of the large solid angle subtended by the scattered X-rays.

**Figure 5:**
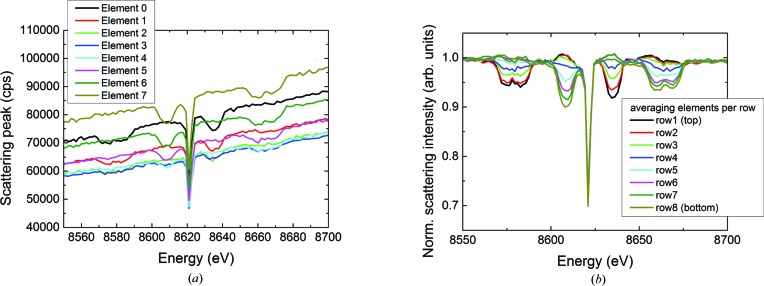
Non-fluorescent scattering from 1 millimolal aqueous nickel nitrate solution in a germanium detector. (*a*) Raw count rates in the first column of pixels. (*b*) Processed data averaged over each row of pixels. The linear energy-dependent variation was subtracted from the raw data in (*a*), and then the remaining counts in each pixel were all normalized to 1 away from the glitches.

**Figure 6:**
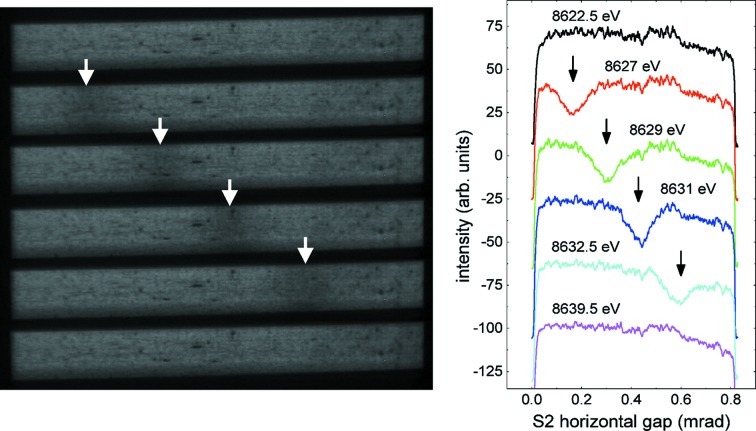
(Left) Stack of beam images collected immediately downstream from the monochromator while the monochromator’s energy setting is scanned through a glitch. (Right) Vertically integrated intensity for each image.

Previous work devoted to understanding the presence of glitches and to improving their normalization in transmission EXAFS measurements has stressed the importance of several critical factors such as harmonic rejection, detector linearity, and sample homogeneity and uniformity in thickness (Stern & Lu, 1982 ▸; Comin *et al.*, 1983 ▸; Bauchspiess & Crozier, 1984 ▸; van Zuylen & van der Hoek, 1986 ▸; Dobson *et al.*, 1989 ▸; Bridges *et al.*, 1991 ▸, 1992 ▸; Li *et al.*, 1994 ▸). All the precautions advised in these papers were addressed in this paper’s measurements. The Rh stripe of the harmonic rejection mirrors was set to a grazing incidence angle of 5 mrad to decrease the harmonic content. Both the ionization chamber and the fluorescence detector were working in the linear regime. The fluorescence detector was operated at approximately 40 kcps, which ensures that the dead time and any other nonlinear behaviors are negligible. The homogeneity of the sample was guaranteed by using a solution loaded into a plastic capillary that was larger than the beam size and was carefully aligned to the beam. The continuing influence of the glitches despite these measures suggests that some additional effect prevents the compensation of glitches in EXAFS measurements performed with multi-element fluorescence detectors. Detailed inspection of the counts collected by each element of the germanium detector revealed that the non-fluorescent scattering changed uniformly over all pixels as long as no glitch was excited, but was altered differently in different rows of pixels when the energy setting entered a glitch, as shown in Fig. 5 ▸. The only exception is the prominent central glitch, which reduces the non-fluorescent scattering uniformly over the whole detector. This alteration has not previously been noted, and it indicates that the polarization of the X-rays reaching the sample is altered at all glitches except the central one. Scintillator images of the X-ray beam immediately downstream from the monochromator and upstream from the focusing mirrors have shown that most of the glitches appear as dark vertical stripes that travel either left or right across the width of the beam as the energy setting is scanned (Fig. 6 ▸). The only exception is again the central glitch in Fig. 5 ▸, at which the intensity drops uniformly over the entire cross section of the beam. The spatial inhomogeneity in this unfocused beam together with variations in the sample thickness were named by Bridges *et al.* (1991 ▸, 1992 ▸) and by Li *et al.* (1994 ▸) as the cause of the glitches observed in transmission EXAFS measurements. These authors described a vertical displacement of the dark band associated with a glitch, the result of the nonzero vertical divergence of the beam incident on their monochromator. Here, by contrast, a horizontal displacement of the dark band with the monochromator’s energy setting is observed. This is the result of the large horizontal divergence of the X-ray beam from the wiggler, which causes different parts of the beam to strike the crystals at different azimuthal angles. Bridges *et al.* (1991 ▸, 1992 ▸) also proposed that the use of two successive double-crystal monochromators in a dispersive configuration would compensate for the glitches appearing because of sample inhomogeneities. This, however, can work only if the beam impinging on the sample is unfocused, for then the dark band produced by the glitch in the second pair of crystals could be made spatially opposite to the one produced by the first pair of crystals. Sample inhomogeneities would then be averaged out by the increased area of the sample covered by the dark bands. This idea would not work here because the sample is a homogeneous solution and receives a focused beam. It has been observed that the focused beam remains spatially homogeneous, without showing any structure but only an overall loss of intensity, when the monochromator is scanned through a glitch. Other authors have suggested increasing the horizontal acceptance angle of the beam to smooth out the glitch structures (van der Laan & Thole, 1988 ▸). This is not always feasible in most beamlines and would in any case only work effectively for those reflections with strong azimuthal dependence, which not all reflections have as will be seen here.

Glitches have long been ascribed to the excitation of additional ‘parasitic’ Bragg reflections along with the principal Bragg reflection. This is represented in reciprocal space by the passage of additional nodes of the crystal’s reciprocal lattice through the Ewald sphere as the crystal is scanned. The loss of intensity reaching the sample at glitches is then easily explained. However, the evidence of a change in the monochromatic beam’s polarization state demands a more thorough understanding of both the real polarization state of the X-rays emerging from the wiggler and the way in which parasitic reflections may change it. This is particularly important when dilute systems are being studied, for their low fluorescence yield requires the detector to be brought very close to the sample, so that each pixel subtends a larger solid angle and is thus more strongly affected by any rotation of the beam polarization. For the former, the ray-tracing program *SHADOW* (Sanchez del Rio *et al.*, 2011 ▸) has been used to calculate the Stokes parameters of a series of rays generated by the wiggler. For the latter, if the space group of a crystal and the quantities 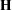, 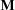 and ϕ that define the crystal orientation are known, then the photon energies at which the Bragg condition is fulfilled simultaneously for the principal reflection 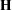 and one or more additional reflections 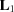, 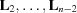 can be calculated by using the Ewald sphere construction. As there are one incident beam and 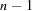 diffracted beams, this is called an *n*-beam diffraction case. Calculations have been performed as described by Rek *et al.* (1984 ▸). In this way the parasitic Bragg reflections that cause each observed glitch can be determined. The subsequent calculation of expected polarization alterations at each glitch has been performed using *n*-beam dynamical diffraction theory. X-ray diffraction from large perfect crystals such as those that make up the monochromator must be treated by using dynamical diffraction in order to account correctly for absorption, extinction and coherent coupling between the incident and diffracted waves within each crystal. Dynamical diffraction calculations for 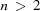 generally must be treated numerically; the publicly available software package *BRL* (Stepanov & Ulyanenkov, 1994*a* ▸,*b* ▸) was used here.

Once the effects of *n*-beam dynamical diffraction from the monochromator crystals are fully understood, a model of the scattering from the sample is required. The fluorescence generally will contain multiple spectral lines of which only one is to be measured. Aside from fluorescence, the processes that contribute to the scattered photon flux collected by the germanium detector are the coherent scattering, which is elastic, and the Compton scattering, which is inelastic. The low-energy tail of the Compton scattering distribution and the incomplete charge collection of real fluorescence detectors can add to the background within the fluorescence peak. This is possible even in the example of this study, where the separation between the elastic scattering peak and the fluorescence peak (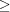
1000 eV) is much larger than the detector’s energy resolution (∼200 eV). For concentrated samples this effect is not critical because the fluorescence yield is much larger than the background, but it is a problem when one tries to measure the low fluorescence yield of dilute samples. To quantify this, both coherent and Compton differential scattering cross sections are calculated here, including their dependence on the polarization of the incident photons, but disregarding the scattered photons’ polarization as this is not measured. Based on these, a fitting procedure has been developed to estimate the degree and type of polarization of the X-rays incident on the sample from all of the pixel readings of the non-fluorescent scattering in the germanium detector.

The outcome will be of interest not only to the EXAFS community but also to those studying diffraction, because it is an experimental observation of how a multiple-beam diffraction case can rotate the polarization of X-rays. It must be stressed that all of the physical phenomena discussed in this article – the polarization of the incident beam, the multiple-beam dynamical diffraction and the photon scattering from the sample – must be understood if the observed effects are to be explained and a solution for normalization at glitches is to be found. In the next three sections, each one of these phenomena will be explained and applied to the I20-scanning wiggler, the I20-scanning crystal monochromator and an aqueous Ni(NO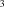)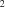 sample solution. Then a polarization-dependent fitting model for the spatial distribution of the sample’s coherent and Compton scattering onto the fluorescence detector will be developed. The model will be applied to a set of measurements of the scattering of a 100 micromolal aqueous solution of 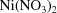 as a function of incident photon energy. The values derived in this way for the polarization state of the incident beam will be compared with the wiggler beam simulations and with multiple-beam dynamical diffraction theory. The insights gained from this study will aid the future development of more accurate normalization procedures.

## 2. Polarization of wiggler beam   

X-ray beams produced by synchrotron sources such as bending magnets and planar insertion devices are strongly linearly polarized in the horizontal direction because the plane of the electron orbit is horizontal. However, perfect linear polarization is observed only for X-rays that lie exactly in the plane of the electron orbit. X-rays that emerge from a bending magnet at a nonzero vertical angle from this plane are elliptically polarized, with the phase difference between the horizontal and vertical components of the electric field being 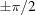 depending on whether the ray is above or below the electron orbit. The X-ray beam from a planar wiggler may be viewed as the incoherent sum of X-rays produced by a row of bending magnets of alternating curvature. A ray emitted at nonzero vertical angle by a wiggler with an even number of periods thus has an equal probability of being left or right elliptically polarized. Hence the incoherent sum of rays emitted at all poles of the wiggler into a given nonzero vertical angle will be partly unpolarized. The polarization of a general electric field 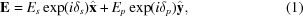
where the magnitudes 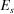, 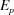 and the phases 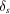, 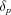 are all real, is described by its Stokes parameters: 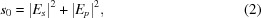


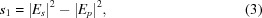


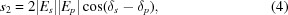


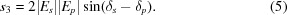
In the ray-tracing program *SHADOW* (Sanchez del Rio *et al.*, 2011 ▸), each ray is monochromatic and has a total intensity 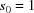. The resulting scatter plots represent each ray with a single dot. 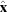 and 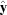 are, respectively, the horizontal and vertical unit vectors orthogonal to the direction of ray propagation. The parameters of the simulation are shown in Table 1 ▸. The scatter plots for 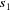 and 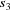 are shown in Figs. 1 and 2 of the supporting information. It was found that 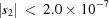 for all rays.

The calculated Stokes parameters may be interpreted as follows:

(i) 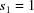 in the plane of the electron orbit. Therefore, as expected, all rays here are completely linearly polarized in the horizontal direction.

(ii) 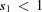 out of the plane of the electron orbit, and it decreases as the vertical angle increases. Therefore, rays emerging at a nonzero vertical angle have not only a horizontal but also a vertical component in their electric fields.

(iii) 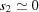 for all rays. Therefore, the vertical component of the electric field of each ray is out of phase with the horizontal component by 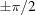.

(iv) 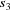 has an equal probability of being positive or negative at a given vertical angle. Therefore, the incoherent sum over all rays at a given nonzero vertical angle results in a depolarization of the beam.

The average value of 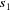 for all 500 000 rays in each simulation is approximately determined by taking a 250 bin histogram. Each bin includes the number of rays within its range of 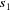. For X-rays of 8540 and 8700 eV, at the beginning and the end of the energy range examined, the average values of 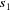 are, respectively, 0.9560 and 0.9558. Thus, respectively, 4.40 and 4.42% of the intensity entering the beamline through the primary slits is expected to be vertically polarized. Neither the window nor the grazing incidence mirrors would significantly change this value before the beam reaches the monochromator.

## 3. Multiple-beam dynamical diffraction theory   

The theory of dynamical diffraction by a perfect crystal begins with the decomposition of the electric displacement 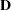 inside the crystal into a Fourier series: 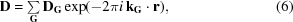
where 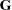 is a reciprocal lattice vector of the crystal. Normally only two terms, 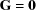 and 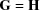, are non-negligible, the other reciprocal lattice vectors being too far away from the Ewald sphere to result in any significant contribution to the electric displacement. (Note that 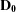 is the incident displacement and 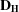 the diffracted displacement.) The two-beam equations for the diffracted displacements, which can be solved analytically, are then derived from Maxwell’s equations and the boundary conditions of the displacement. Standard treatments of this have been given by Zachariasen (1945 ▸), Batterman & Cole (1964 ▸) and Authier (1970 ▸). In the most commonly encountered two-beam case, in which the reciprocal lattice vector 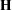 lies in the plane of 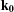 and the unit surface normal 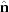, a distinction is made between incident beam polarization parallel to the crystal surface (*s*-polarization) and incident beam polarization in the plane perpendicular to the crystal surface (*p*-polarization). It is found that *s*-polarized X-rays remain *s*-polarized after diffraction and likewise that *p*-polarized X-rays remain *p*-polarized. However, the rocking curve for *p*-polarized X-rays is narrower and has a lower peak intensity than that for *s*-polarized X-rays. Therefore, even this common case will alter the X-ray polarization if the incident beam is not purely *s*- or *p*-polarized. This effect is especially significant if the Bragg angle is 45°, for there the diffracted intensity for the *p*-polarized beam drops to zero, making the crystal into an X-ray polarizer.

If the number *n* of reciprocal lattice vectors close to the Ewald sphere is greater than 2, the equations that determine the amplitudes and phases of all the diffracted beams are still derived from Maxwell’s equations and the boundary conditions, but in general they can no longer be solved analytically. They can, however, still be solved numerically. Colella (1974 ▸), Kohn (1979 ▸), Stepanov & Ulyanenkov (1994*a* ▸) and Stetsko & Chang (1997 ▸) have all treated this problem, paying special attention to the particularly demanding cases of grazing incidence and diffraction (which will not appear in this paper). The coherent coupling among the *n* waves within the crystal generally causes very complex effects, including the well known *Umweganregung* (‘detour excitation’) (Renninger, 1937 ▸), in which the diffracted wave from a nominally forbidden reflection such as 222 in diamond or silicon is greatly increased by the simultaneous excitation of an allowed reflection. Moreover, if one of the additional reciprocal lattice vectors 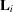 does not lie in the plane spanned by 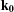 and 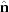, then the diffracted wave produced by 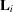 may have mixed polarization even if the incident beam is purely *s*- or *p*-polarized. Through coupling between the 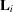 and 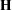 waves inside the crystal, the diffracted wave from 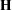 may acquire a mixture of *s*- and *p*-polarization as well.

**Figure 7:**
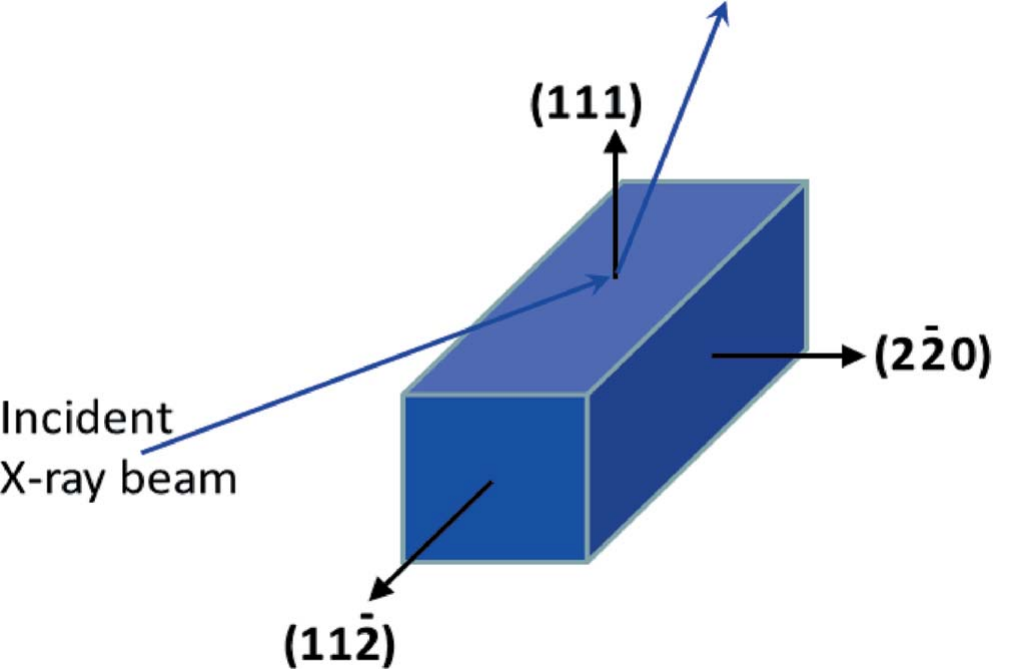
Ideal lattice planes of monochromator crystal surfaces. ϕ should be 0° from 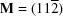.

**Figure 8 fig8:**
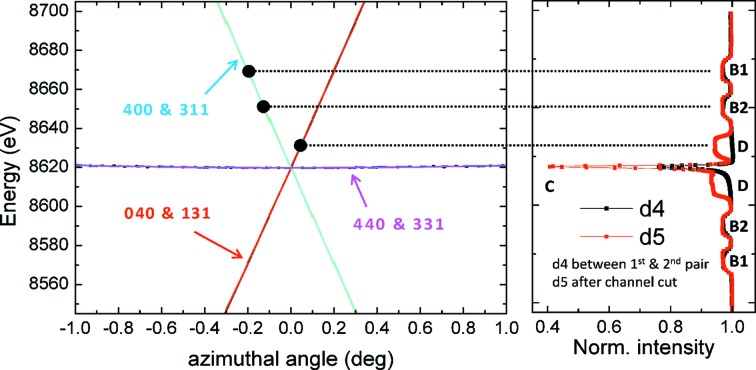
(Left) Plot of energy *versus* azimuthal angle ϕ at which parasitic Bragg reflections can be excited on the I20-scanning four-bounce monochromator in the neighborhood of the Ni *K* edge. The plot was generated by software provided by one of the authors (Bowron, 2012 ▸). The software was based on the work of Rek *et al.* (1984 ▸). Each trace is labeled with its parasitic Bragg reflections. (Right) Experimental data showing glitches measured by a diode detector D4 between the first and second pair of bounces and by a diode detector D5 just downstream from the monochromator. The label for each glitch is on the far right. The solid circles on the left, which are connected by horizontal dotted lines to the corresponding measured glitches on the right, show the estimated true azimuthal angle ϕ of the crystal that produces each glitch (see Table 2 ▸).

The nominal lattice orientation of the crystal surfaces is shown in Fig. 7 ▸. The incident beam will lie approximately within the plane spanned by 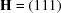 and 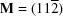. More precise measurements of the true azimuthal orientation ϕ for each crystal can be derived by comparing the measured energy settings of the monochromator at which glitches appear with a theoretical calculation of the multiple-beam cases. This process is shown in Fig. 8 for energies slightly above the Ni *K* absorption edge at 8333 eV (Center for X-ray Optics & Advanced Light Source, 2009 ▸). The particular crystal that is responsible for any glitch was determined as follows:

(*a*) With a diode detector after the second crystal but before the channel cut, the position of each glitch was checked at different settings of the second crystal roll. If the glitch’s position did not change, the glitch arose from the first crystal. If it did, the glitch arose from the second crystal.

(*b*) With no detector between the crystals but with one downstream from the channel cut, the glitches were measured again. Any new glitches arose from the channel cut.

All glitches could be traced back to a single crystal except the strong narrow central one, which was created by all crystals because of the insensitivity of the 440–311 multiple-beam case to the azimuthal angle. The results are listed in Table 2 ▸.

**Figure 9:**
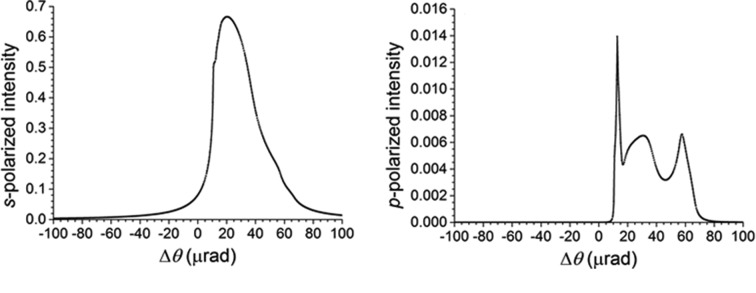
Rocking curves of Si 111 as calculated by *BRL* (Stepanov & Ulyanenkov, 1994*b* ▸) when either the parasitic reflections 400 and 311, or the parasitic reflections 040 and 131, are also excited. The incident beam polarization is *s* (parallel to the crystal surface). The *s*-polarized component of the intensity diffracted into the 111 reflection is shown in (*a*); the *p*-polarized component is shown in (*b*).

**Figure 10:**
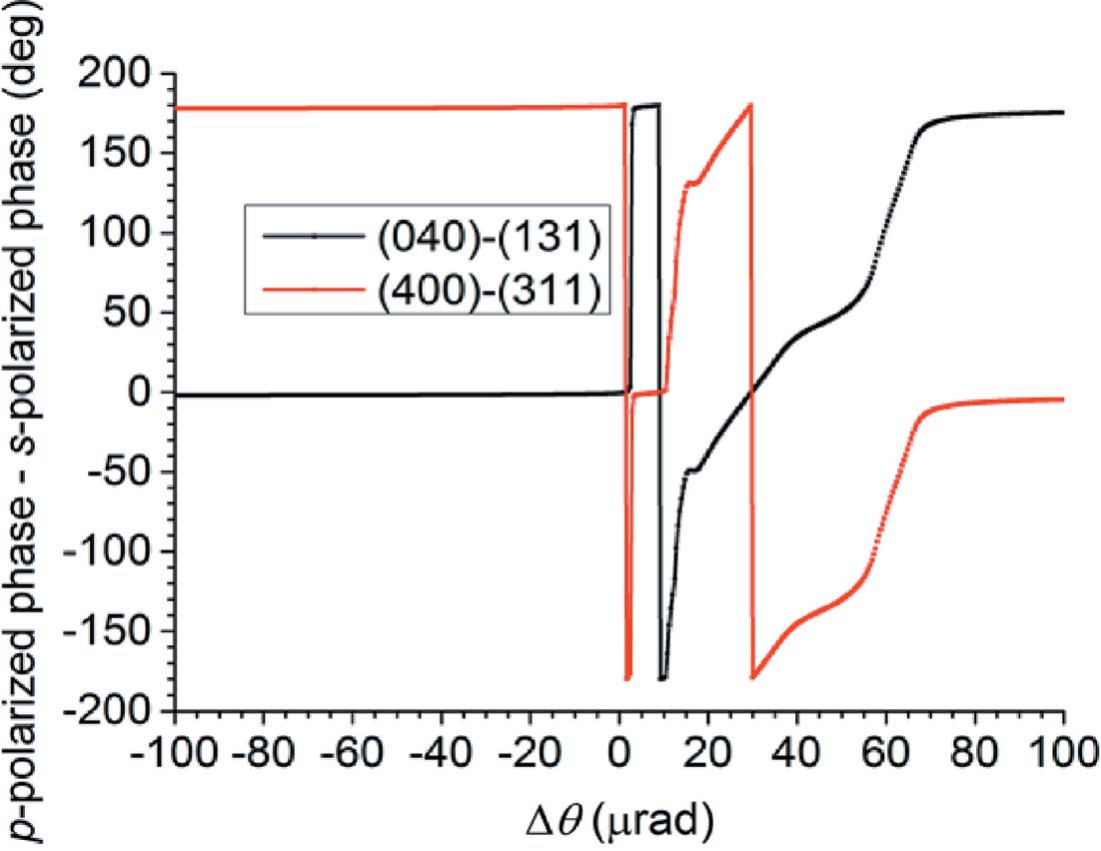
Phase difference between *s*- and *p*-polarized amplitudes of the wave diffracted into the 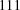 direction when the parasitic reflections 400 and 311, or 040 and 131, are excited.

**Figure 11:**
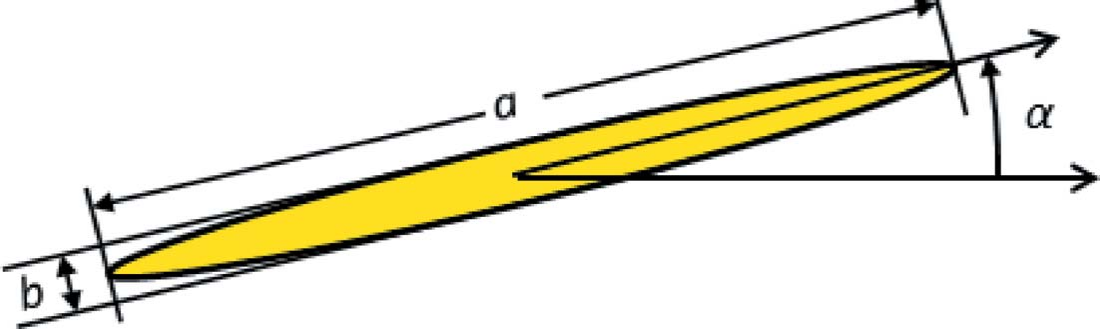
Definitions of major axis *a*, minor axis *b* and inclination angle α from the horizontal for the polarization ellipse.

*BRL* (Stepanov & Ulyanenkov, 1994*b* ▸) is now used to calculate the amplitude and phase of the beam diffracted by a silicon crystal into the 111 reflection when the parasitic reflections 400 and 311, or the parasitic reflections 040 and 131, are excited. In the following, the incident beam is assumed to be a plane wave of which the direction of propagation is scanned through an angle 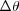 from the Bragg angle for Si 111. As an example, the photon energy is chosen as 8650 eV, the measured energy of glitch B2 in Fig. 8 ▸. The incident beam’s polarization is assumed to be linear horizontal, corresponding to the *s*-polarization state. The rocking curves are shown in Fig. 9 ▸(*a*) for the *s*-polarized component of the intensity diffracted into the 111 direction and in Fig. 9 ▸(*b*) for the *p*-polarized component of this intensity. Both sets of parasitic reflections under consideration yield the same rocking curves because of the high lattice symmetry. Note the small – but still significant – *p*-polarized component that has appeared because of the excitation of the parasitic reflections. A calculation of the phase difference between the *p*-polarized and the *s*-polarized com­ponents of the wave diffracted into the 111 direction is shown in Fig. 10 ▸. Note that the two four-beam cases produce phase differences that are 180° apart. The 111 diffracted beam is thus elliptically polarized wherever the parasitic Bragg reflections have significant strength, with the helicity depending on which four-beam case is excited. The polarization ellipse is described by its major axis *a*, its minor axis *b* and its inclination angle α from the horizontal, as shown in Fig. 11 ▸. If the *s*-polarized electric field amplitude is given by 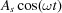 and the *p*-polarized electric field amplitude by 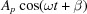, then the instantaneous Poynting vector of the total field sweeps out an ellipse with 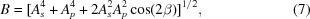


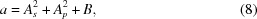


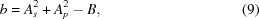


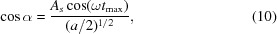


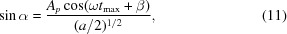
where 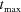 is determined by the relations 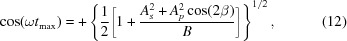


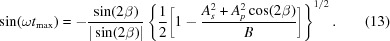



**Figure 12:**
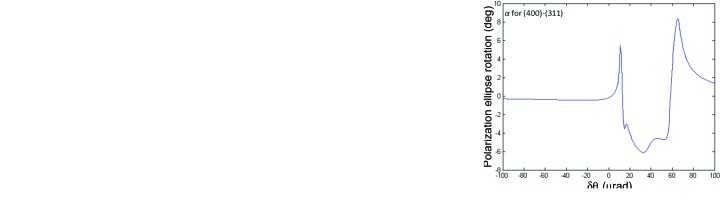
(*a*) Ratio 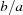 of minor axis to major axis of the polarization ellipse of the 111 diffracted wave when either the 400 and 311 reflections or the 040 and 131 reflections are excited. (*b*) Inclination angle α from the horizontal of the polarization ellipse of the 111 diffracted wave when the 040 and 131 reflections are excited. (*c*) Same as (*b*) but with the 400 and 311 reflections excited. All calculations performed by *BRL* (Stepanov & Ulyanenkov, 1994*b* ▸) as a function of deviation 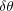 from the Bragg angle of the 111 reflection.

The parameters of the polarization ellipse of the 111 diffracted wave when either the 400 and 311 reflections or the 040 and 131 reflections are excited are displayed as a function of the deviation from the Bragg angle of 111 in Fig. 12 ▸. The 111 diffracted wave remains nearly linearly polarized because 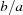 is small, but its polarization is rotated from the horizontal by 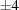
–6°. By contrast, at the central glitch, which is excited by the 440 and 331 reflections, *BRL* calculates a 111 *p*-polarized intensity less than 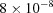, so that here the 111 diffracted wave remains entirely *s*-polarized. This confirms what could have been guessed by symmetry, since the 440 and 331 reciprocal lattice vectors lie exactly in the plane of diffraction and therefore would not allow the introduction of a symmetry-breaking elliptical polarization to the 111 diffracted wave.

To conclude this section, a few words may be said about the widths of the glitches in Fig. 8 ▸, and in particular about why the central glitch is so much narrower than all the others. Images of the X-ray beam taken immediately downstream from the monochromator show that glitches B1, B2 and D appear as a dark line crossing from left to right or from right to left across the beam as the energy setting is scanned. Therefore, the widths of these glitches in the diode detectors are determined by the horizontal opening angle of the beam. In the central glitch, however, the entire beam darkens at once and then brightens at once within a very narrow range. The width of this glitch is therefore independent of the beam’s horizontal opening angle. In these measurements the horizontal divergence of the wiggler beam is very large (800 µrad) and functions as an effective variation of the azimuthal angle across the horizontal width of the X-ray beam. If one imagines a window covering a narrow range of energies (approximately equal to the 111 Darwin width) but a wide 800 µrad (0.046°) range of azimuthal angles in the left-hand plot of Fig. 8 ▸, and shifts this window along this plot’s energy axis, the behavior described in this paragraph becomes clear. In glitches B1, B2 and D, only a small part of the wiggler beam falls on the crystal at the correct azimuthal angle to excite one of the multiple-beam cases, and this part appears at different azimuthal angles (different horizontal positions in the beam) as the energy setting is scanned. On the other hand, in the central glitch the entire wiggler beam can lie within the range of azimuthal angles at which the 440 and 331 reflections are excited.

## 4. Compton and coherent scattering from sample   

As shown in Fig. 1 ▸, the monochromated X-ray beam is focused horizontally by a sagittally curved cylindrical mirror and vertically by a meridionally mechanically bent mirror. After reflection from two flat harmonic rejection mirrors, the beam finally converges to its focus on the sample. None of these mirrors will significantly affect the polarization of the X-rays. The sample scatters a portion of the X-rays into the large-area multi-element fluorescence detector as shown in Fig. 2 ▸. The experimental data to be presented from this section onwards were collected with the horizontal width of the slit placed immediately downstream from the monochromator (not shown in Fig. 1 ▸) narrowed to 0.067 mrad. This is about equal to the horizontal width of the dark lines introduced by the glitches into the beam emerging from the monochromator, and ensures that when a glitch is present few X-rays reach the sample that do not excite one of the four-beam cases in Fig. 8 ▸. The sample was a 100 micromolal aqueous solution of 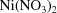 contained in a MicroRT polymer capillary of 25 µm wall thickness and 2 mm diameter (purchased from MiTeGen) oriented in the horizontal plane 45° from the beam. Because the polymer consists chiefly of carbon and hydrogen atoms that have very small X-ray scattering cross sections, and because the walls of the capillary are so thin, it can reasonably be assumed that the scattering from the capillary is negligible. The footprint of the focused beam on the sample is approximately 0.5 × 0.5 mm, and the beam crosses a length of approximately 2.8 mm through the sample solution. As this is a significant fraction of the 5 mm width of one pixel on the detector, it would not in principle be correct to treat the sample as a point. Furthermore, because the attenuation length of the X-rays in liquid water is only 1.2 mm, attenuation will have a significant effect on the photon flux scattered by the sample. Unfortunately, an exact calculation of the effect of attenuation and multiple scattering on the photon flux reaching each pixel of the detector would scarcely be feasible. It would require a three-dimensional integration of a double trajectory (incident surface of sample solution to scattering volume element, then scattering volume element to exit surface facing a given detector pixel) over the whole sample volume. The results would be strongly dependent on the exact sample shape and could be calculated only for a few very simple cases. However, one expects attenuation and multiple scattering to affect the photon count rates in the detector much more strongly along the direction of the beam than along the vertical direction (Chantler *et al.*, 2012 ▸). This can be easily illustrated by performing a calculation of the attenuation of the elastically scattered photons viewed at different angles in the detector. For simplicity we have calculated the attenuation of 8621.5 eV photons entering and leaving a cylindrical sample oriented at 45° with respect to the incident beam. Owing to the photons’ different path lengths, the upstream pixels receive higher count rates than the downstream ones, but there is little difference between pixels in the same column. (See Fig. 3 of the supporting information.) The coherent and Compton scattering processes mentioned in §1, on the other hand, are affected most strongly in the vertical direction if the polarization of the incident beam is rotated. Therefore, even without an accurate treatment of attenuation and multiple scattering, one may use the observed change in the vertical distribution of photon flux in the presence of a glitch to accurately determine the angle of the polarization rotation.

Because the 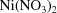 solute is so dilute, the coherent and Compton scattering from the 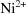 cations and the 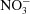 anions is neglected. Only the scattering from the water molecules will be considered. The coherent scattering is always elastic, whereas the Compton scattering is inelastic. If a photon undergoes Compton scattering by an electron at rest through a deflection angle Θ, the ratio of the energy 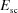 of the scattered photon to the energy 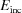 of the incident photon is 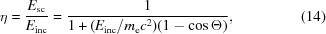
where *m*_e_ is the mass of the electron and *c* is the speed of light. The incident X-ray photon energy was scanned from 8545 to 8700 eV. The pulses generated in the multi-element fluorescence detector by the incidence of an X-ray photon increase in height with the incident photon energy. The pulses are binned according to their height, and the number of pulses in each bin is plotted against the number of each bin as in Fig. 2 ▸. The scattering data that will be analyzed in this article are sums over all photons striking the detector with energies from 7880 to 9250 eV, thus excluding the Ni *K*α fluorescence. Under these circumstances, all photons scattered into the multi-element fluorescence detector by either the coherent or the Compton process have energies within this window. Therefore the two processes both contribute to the scattering peak in Fig. 2 ▸ and cannot be separated. The Ni *K*β emission line at 8265 eV (Center for X-ray Optics & Advanced Light Source, 2009 ▸) also falls within this energy window. However, the Ni *K*β emission line is almost one order of magnitude less intense than the Ni *K*α doublet. The Ni *K*β line contributed only 90 counts per second per pixel as opposed to the 40 000 measured in the scattering peak. Hence the Ni *K*β emission line can be neglected.

**Figure 13:**
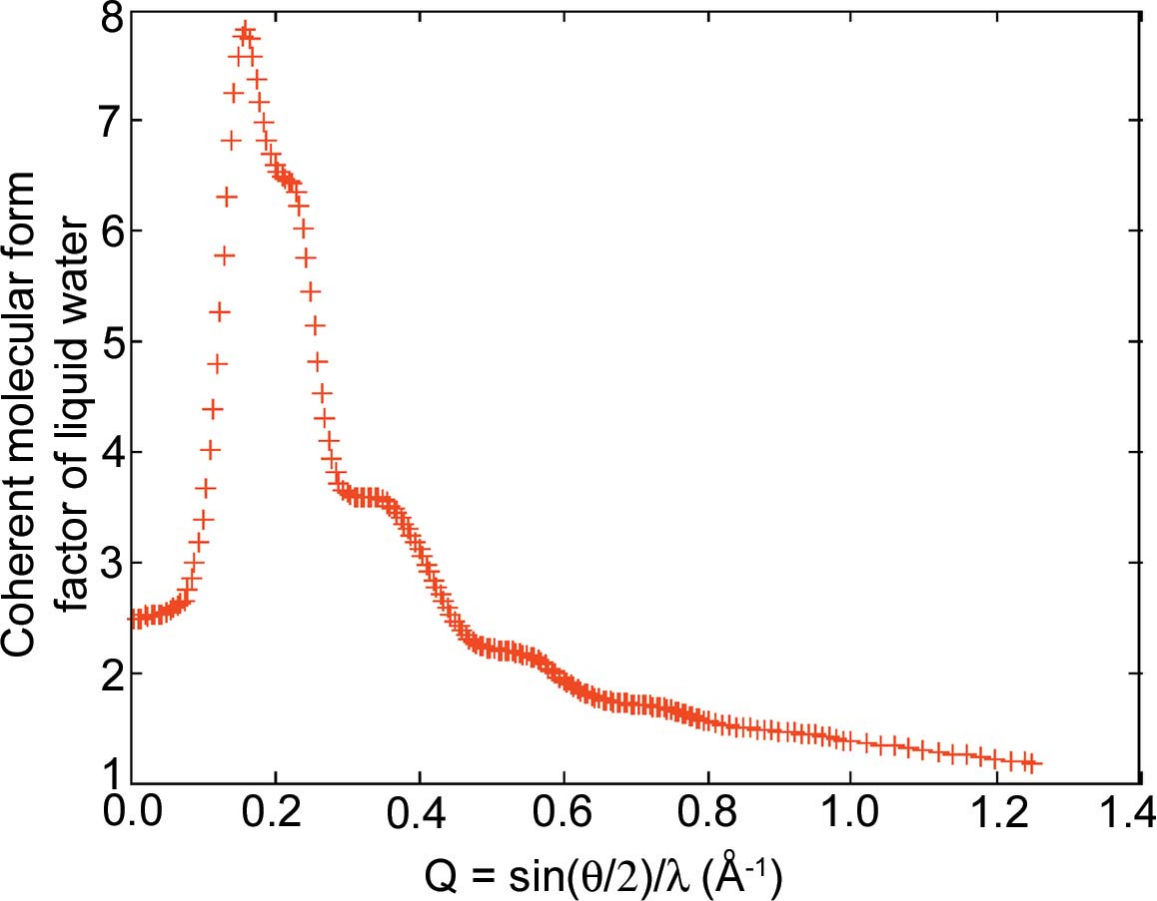
Coherent molecular form factor of liquid water at 293 K as a function of scattering vector. θ is the deflection angle of the scattering and λ is the X-ray wavelength.

The coherent form factors per molecule in liquid water were interpolated from tabulated data published by Morin (1982 ▸) at 293 K. A set of values is plotted in Fig. 13 ▸. For a single free water molecule, the form factor at 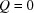 would be equal to ten, the number of electrons 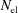 in the molecule, but in liquid water the form factor per molecule is less than this because of intermolecular interference effects. If the unit vector directed from the scattering point to the observation point is 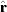, if the X-ray beam at the scatterer is linearly polarized along the unit vector 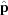, if Θ is the deflection angle of the scattering and if 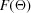 is the coherent form factor per molecule, then the differential coherent scattering cross section is given by 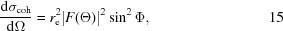
where 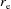 is the classical radius of the electron, 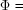 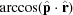 and 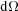 is the differential solid angle.

The differential Compton scattering cross section is given by the Klein–Nishina formula (Klein & Nishina, 1929 ▸) for a single electron. Because Compton scattering is incoherent, the differential Compton scattering cross section of one water molecule will be approximately 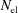 times that of a free electron.^1^ If the incident photon is linearly polarized along 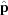 but the detector is insensitive to the scattered photon’s polarization, then the differential Compton scattering cross section is 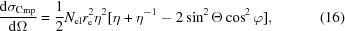
where φ is the angle between 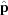 and the scattering plane.

Because the incident X-ray beam on the sample will have a small vertically polarized component along with the principal horizontally polarized component (see §2), Fig. 4 of the supporting information displays the differential coherent and Compton scattering cross sections of 8545 eV incident photons of both polarizations as a function of scattering angle Θ and azimuthal angle Φ across the surface of the multi-element fluorescence detector as calculated using equations (15) and (16). Similar calculated cross sections were obtained for 8700 eV incident photons.

## 5. Fitting model for scattered flux   

On the basis of the discussions in §§[Sec sec2]2–4 [Sec sec4], a simple fitting model for the spatial distribution of the photon flux scattered into the multi-element fluorescence detector has been created. To account for the possibility that the sample is not precisely centered on the multi-element fluorescence detector, 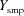 and 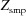 were defined as the sample’s misalignment in the vertical direction and along the incident beam, respectively. The distance *D* from the sample to the multi-element fluorescence detector was 110 mm. The unit vector 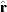 originates at the sample at 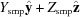 and points toward some position 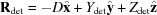 on the detector. 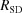 is the distance between the sample and the selected point on the detector, and 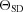 is the corresponding deflection angle of scattering. The coordinates 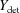 and 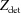 lie on a mesh of 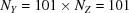 points covering the entire 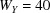
 mm × 
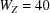
 mm surface of the detector. The relevant equations are 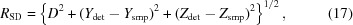


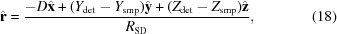


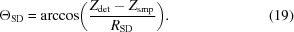
Now, if the glitches introduce a polarization rotation ξ, the major and minor polarization vectors 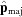 and 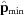 are given by 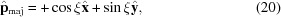


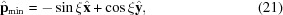
and the corresponding azimuthal angles 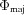 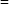 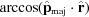 and 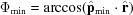 are calculated for coherent scattering. For Compton scattering, the unit normal to the scattering plane is 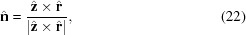
and from this the azimuthal vectors 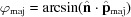 and 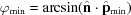 are calculated. The ratio of the incident intensity with minor polarization to that with major polarization is defined as *M*. Finally, the obliquity factor 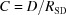 and the area 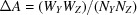 per detector point are determined. If a scale factor *S* is defined to match the theoretical calculation of the cross sections to the experimental data, then at last the power scattered into the small element 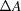 on the surface of the detector around 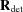 is 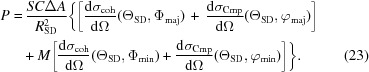
Indexing the points on the detector with 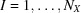 and 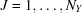, one determines the approximate total power scattered into each pixel *K* on the detector: 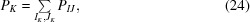
where 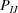 is calculated at point *I*, *J* in equation (23), and 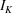, 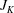 are all values of *I* and *J*, respectively, that refer to a point on the detector lying within pixel *K*.

In the following, all fits using this model were performed by MATLAB (The MathWorks, 2004 ▸).

## 6. Results and discussion   

**Figure 14:**
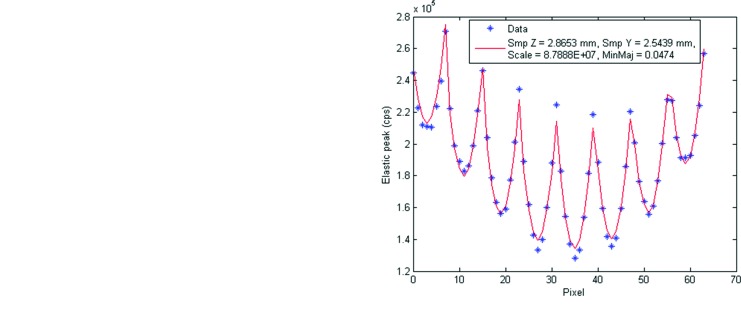
Multi-element detector reading (plus symbols) *versus* fit (solid line) using 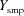 (‘Smp Y’), 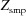 (‘Smp Z’), *S* (‘Scale’) and *M* (‘MinMaj’). The polarization rotation angle ξ is fixed at zero. (*a*) 8545 eV, (*b*) 8700 eV. The configuration of the pixels is shown in Fig. 2 ▸.

**Figure 15:**
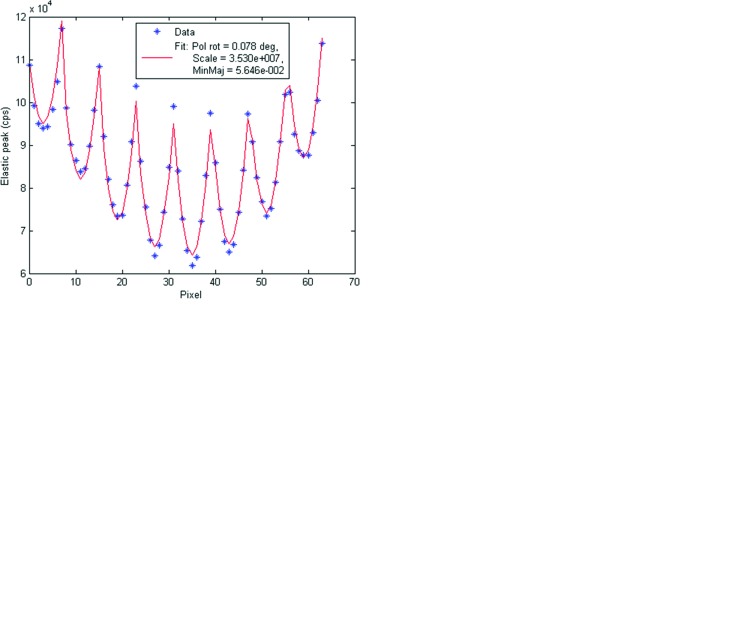
Multi-element detector reading (plus symbols) *versus* fit (solid line) using ξ (‘Pol rot’), *S* (‘Scale’) and *M* (‘MinMaj’). (*a*) Central glitch at 8621.4 eV, (*b*) top of glitch B1 at 8555.4 eV, (*c*) bottom of glitch B1 at 8556.6 eV, (*d*) center of glitch D at 8590.8 eV. The configuration of the pixels is shown in Fig. 2 ▸. See Fig. 8 ▸ for labeling of the glitches.

**Figure 16:**
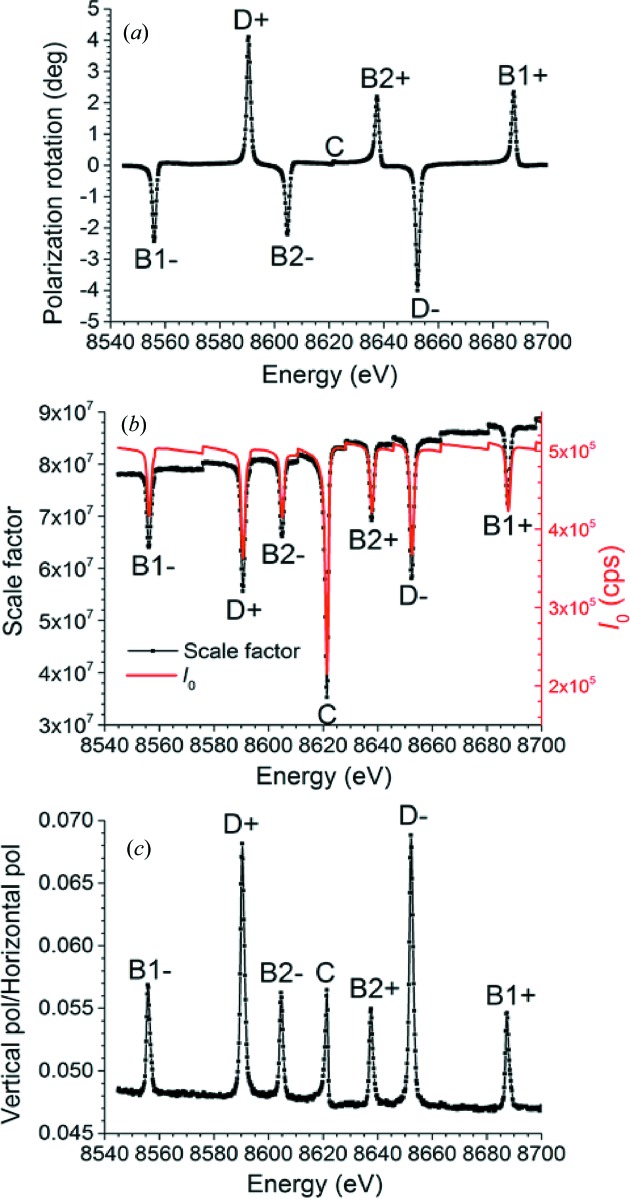
Best-fit parameters (*a*) ξ and (*b*) *S* (with the ionization chamber reading 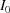 of the incident beam provided for comparison), and (*c*) *M* at all measured incident photon energies.

First, the experimental data at the two ends of the energy scanning range, 8545 and 8700 eV, were fitted to the model in §[Sec sec5]5. Because no glitches were present at these energies, the polarization rotation angle ξ was fixed at zero. Thus the four fitting parameters were 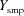, 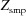, *S* and *M*. The results are shown in Fig. 14 ▸. Note that the best-fit values of *M*, 4.82 and 4.74%, agree well with the calculation of 4.4% in §2. Also note that both fits yield consistent values of 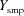 and 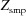. The average of the two values for each parameter, 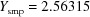
 mm and 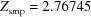
 mm, were then used as fixed values in the next set of fits, of which several examples are displayed in Fig. 15 ▸. These fits were performed at all measured incident photon energies using just three free parameters: ξ, *S* and *M*. Note that the change in vertical dependence of the scattered flux at the glitches is clearly visible as an alteration of the symmetry of the pixel readings in each column. The best-fit values for all fits are shown in Fig. 16 ▸. Several observations can be made:

(1) The maximum polarization rotations ξ at most of the glitches are slightly below the 4–6° range predicted in §3. However, the largest rotation, 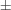
4.3° for the D glitches, falls comfortably within this range. The slight discrepancy may be attributed to some remaining X-rays outside the glitch entering the beamline through the horizontal slits, so that narrower horizontal slits would produce a better match with theory.

(2) The lack of polarization rotation at the central glitch, predicted in §3, is confirmed.

(3) B1, B2 and D glitches viewed on opposite sides of the central glitch rotate the polarization in opposite directions. This also fulfills the prediction made in §3, since two such glitches are created by the two distinct but symmetrically related four-beam cases in which the 400–311 and 040–131 Bragg reflections are excited. See Fig. 12 ▸, which shows that these two four-beam cases do indeed rotate the polarization in opposite directions.

(4) The scale factor *S* increases over time except at the glitches, and it shows several steps. The first effect could not have been caused by drifts in the monochromator, as the monitor reading 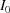 of the incident beam intensity was very stable. Possible causes are the reduction in air absorption around the sample and increasing penetration into the sample as the photon energy increases. The second is due to top-ups of the electron beam current in the storage ring, which occurred every 600 s.

(5) *M* rises slightly but noticeably at every glitch, including the central one. The calculations of §3 do predict a slight ellipticity in the polarization when a glitch other than the central one is excited (see Fig. 12 ▸). However, the fact that the central glitch also appears to introduce a slight ellipticity is probably due to roll misalignments of one or more of the monochromator crystals. Such a misalignment would break the symmetry that would ideally exist when all three Bragg reflections 111, 440 and 331 lie in the diffracting plane.

(6) Away from the glitches, *M* runs from 4.84 to 4.72%, slightly above but still in good agreement with the theoretical calculation of the wiggler beam depolarization in §2.

## 7. Conclusions   

Fluorescence EXAFS data measured on an extremely dilute aqueous solution of 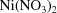 are accompanied by a high level of coherent and Compton scattering from the water. A large-area multi-element fluorescence detector that was used to measure the fluorescence yield also revealed that the spatial dependence of the coherent and Compton scattering was significantly altered in the presence of a monochromator glitch. Furthermore, significant scattered intensity was seen even in the central pixels, where it should have dropped to almost zero if the X-ray beam incident on the sample had been fully horizontally polarized as is generally assumed. The results of this paper show that all of these effects can be explained by a full understanding of the polarization properties of the wiggler beam, the polarization-altering properties of the multiple-beam dynamical diffraction cases that cause the glitches, and the polarization dependence of the coherent and Compton scattering processes. Based on this, a model of the scattering from the sample, although greatly simplified, was developed and used to fit the experimental data with just three physically significant parameters: the polarization rotation, the ratio of intensity polarized in the minor and major directions (respectively, vertical and horizontal in the absence of a glitch), and a scaling factor to match the theoretical calculation to the measured data. The best-fit values for the experimental data were consistent with theory. Moreover, no additional fitting parameter for background needed to be introduced, the treatment of the vertical polarization of the incident beam being sufficient to account for the large intensity remaining at the center of the multi-element fluorescence detector.

Evidently, an improved procedure for normalizing the fluorescence yield of very dilute solutions must account for the change in spatial distribution of the coherent and Compton scattering into the detector when a glitch is encountered. This article has shown how such a change can be explained and measured so that more accurate normalization methods can be derived. With some additional work, the methods of this study could be written as a software package capable of predicting glitch positions and polarization states over energy ranges surrounding a variety of absorption edges. The polarization properties of the X-ray source, the crystal monochromator and the sample scattering could each be treated as a separate module independent of the other two. In this way, other synchrotron X-ray sources such as undulators and bending magnets, and sample matrices other than water, could also be modeled. The procedure explained in this article will thus be applicable to many other beamlines and sample systems besides the particular ones that have here been examined.

## Supplementary Material

Figures for scatter plots. DOI: 10.1107/S1600576716009183/te5014sup1.pdf

## Figures and Tables

**Table 1:** Parameters for the *SHADOW* ray-tracing simulations of the polarization state of the I20-scanning wiggler

Electron energy	3.0 GeV
R.m.s. horizontal electron beam width	121.62 µm
R.m.s. vertical electron beam width	3.56 µm
Horizontal emittance	2.6 nm rad
Vertical emittance	8.0 pm rad
Wiggler upstream from electron beam waist by	7.5 cm
Number of periods in wiggler	24
Length of wiggler period	83 mm
Wiggler gap	20 mm
Wiggler deflection parameter	9.303
Wiggler magnetic field	1.2 T
Horizontal primary slit width	0.67 mrad
Vertical primary slit width	0.11 mrad
Number of simulated rays	500 000

**Table 2:** Crystal and parasitic Bragg reflections that cause each glitch, along with the estimated true azimuthal angle of the crystal The first three glitches listed here are those on the high-energy side of the central glitch C. For the corresponding glitches on the low-energy side, the 040 and 400 reflections are switched, as are the 131 and 311 reflections.

Glitch	Crystal	Reflections	True ϕ
B1	1st	400 & 311	−0.20°
B2	2nd	400 & 311	−0.16°
D	Channel cut	040 & 131	+0.06°
C	All	440 & 331	Insensitive

## Footnotes

1 The binding energies of the electrons in the water molecule have here been neglected. In fact, the energy loss through Compton scattering from a free electron is calculated to be 120–170 eV for X-rays entering the multi-element fluorescence detector. This considerably exceeds the binding energies in liquid water of the eight electrons in the molecular orbitals  (30.90 eV),  (17.34 eV),  (13.50 eV) and  (11.16 eV) (Winter *et al.*, 2004 ▸). Hence these electrons contribute the majority of the Compton scattering. The remaining two electrons lie in the  molecular orbital, which is composed mainly of the 1*s* electrons of the oxygen atom. Their binding energy of 543.1 eV (Center for X-ray Optics & Advanced Light Source, 2009 ▸), being much greater than the energy transfer, greatly reduces their contribution to the Compton scattering. However, because the time-averaged distribution of electrons in liquid water will be isotropic, the angular dependence of the observed differential Compton scattering cross section will not be affected by the anisotropy of the bonds within each water molecule. Also, note in Fig. 4 of the supporting information that the angular dependences of both scattering cross sections for horizontally polarized incident photons, which are in the great majority, are very similar. Therefore the angular dependence of the total scattered flux will not be sensitive to the fact that not all electrons in each water molecule contribute equally to the Compton scattering as assumed here.
