# Supplementary material for: Rotation of X-ray polarization in the glitches of a silicon crystal monochromator
Source: J Appl Crystallogr. 2016 Jul 6;49(Pt 4):1209–22. doi: 10.1107/S1600576716009183 (PMC4970495; doi:10.1107/S1600576716009183)
Supplement: Supplementary file 1 [file j-49-01209-sup1.pdf]

Rotation of X-ray polarization in the glitches of a silicon crystal monochromator: Supplemental material

John P. Sutter,[a]* Roberto Boada,[a] Daniel T. Bowron,[b] Sergey A. Stepanov[c] and Sofía Díaz-Moreno[a]

[a]*Diamond Light Source Ltd, Harwell Science and Innovation Campus, Didcot, Oxfordshire OX11 0DE, United Kingdom,* [b]*ISIS Neutron and Muon Source, Rutherford Appleton Laboratory, Didcot, Oxfordshire OX11 0QX, United Kingdom, and* [c]*Advanced Photon Source, Argonne National Laboratory, Argonne, Illinois 60439, USA. E-mail: john.sutter@diamond.ac.uk*

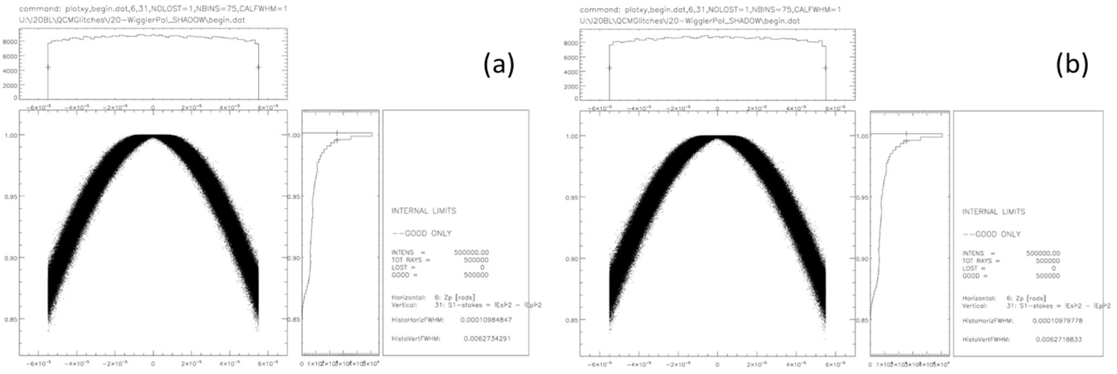

Fig. 1. Scatter plots generated by SHADOW for the Stokes parameter s_1 at (a) 8540 eV (b) 8700 eV. The horizontal angle of each plot is the vertical angle “Zp.”

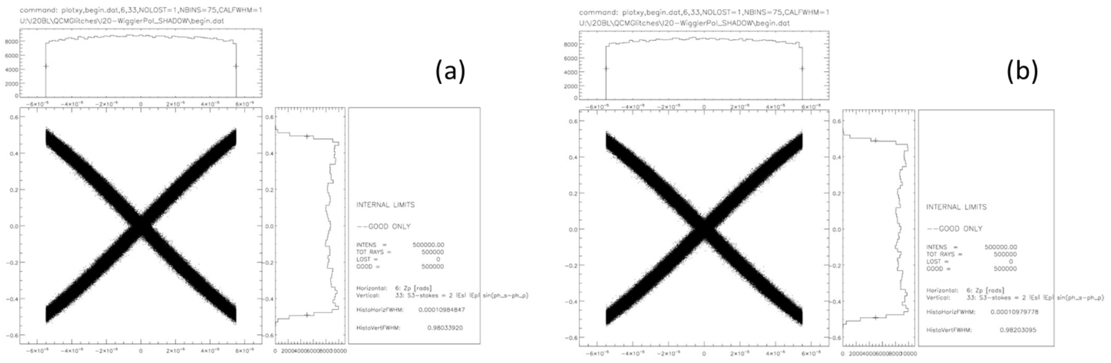

Fig. 2. Scatter plots generated by SHADOW for the Stokes parameter s_3 at (a) 8540 eV (b) 8700 eV. The horizontal angle of each plot is the vertical angle "Zp."

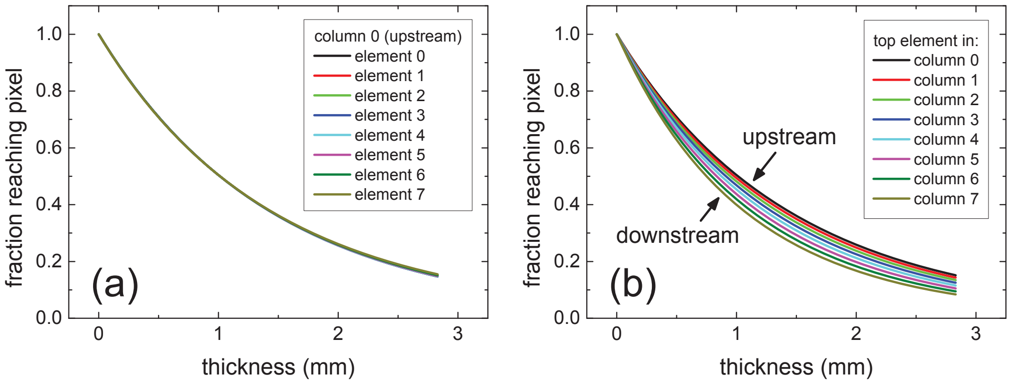

Fig. 3. Estimated fraction of the elastically scattered photons reaching each element of the detector after scattering at specified thickness within the sample: (a) comparison of the elements belonging to the most upstream column, and (b) comparison of the top element of each column in the detector.

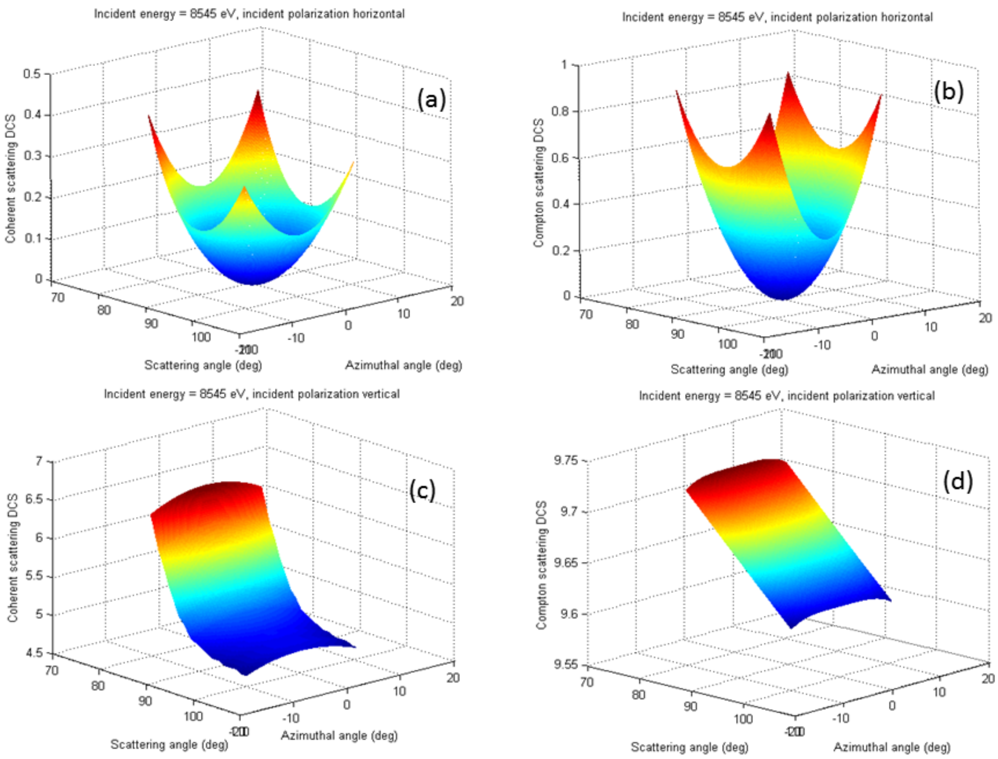

Fig. 4. Differential scattering cross section at 8545 eV incident energy in units of r_e^2 over the surface of the multi-element fluorescence detector for (a) coherent scattering of horizontally polarized photons (b) Compton scattering of horizontally polarized photons (c) coherent scattering of vertically polarized photons (d) Compton scattering of vertically polarized photons. The azimuthal angle in all figures is Φ, the angle between the polarization vector and the observation point.
